# In vitro diagnostics of drug hypersensitivity reactions 

**DOI:** 10.5414/ALX02627E

**Published:** 2026-09-10

**Authors:** Christian Möbs, Sophie C. Hermann Lang, Valerie Härtel, Wolfgang Pfützner

**Affiliations:** 1 Clinical & Experimental Allergology, Department of Dermatology and Allergology, Philipps-Universität Marburg, and; 2 Allergy Section, Department of Dermatology and Allergology, Allergy Center Hesse, University Hospital Marburg, Marburg, Germany

**Keywords:** drug hypersensitivity reaction, allergy, in vitro diagnostics, immediate-type allergy, late-type allergy, IgE, T cells, basophil activation test, lymphocyte transformation test, ELISPOT assay

## Abstract

In vitro diagnostics of drug hypersensitivity reactions comprises different tests that can add important information for identifying the culprit drug. Depending on the patients’ clinical history and underlying immune mechanisms, these tests can be classified into immediate-type and late-type in vitro assays. While they are currently mostly not part of routine diagnostics, knowledge of when to apply which test and what distinct benefits and specific limitations each assay carries, can be helpful when considering the referral of patients with adverse drug reactions for appropriate further testing to specialized centers.

## Introduction 

Drug hypersensitivity reactions (DHR) can be classified into allergic forms, caused by aberrant immune reactions of the adaptive immune system (mainly mediated by antibodies and effector cells like basophils and mast cells or by cytokine-secreting T cells), and into other immunological or non-immunological reactions [1]. Clinical pictures encompass immediate-type DHR like generalized urticaria, angioedema, or systemic anaphylaxis and late-type DHR like rashes or severe cutaneous reactions including Stevens-Johnson syndrome (SJS) or toxic-epidermal necrolysis (TEN). While skin tests are in general the first diagnostic measurement, in vitro diagnostics can provide important additional information and support the identification of culprit drugs, especially since provocation tests are time-consuming, costly, and carry the risk of potentially life-threatening reactions [2]. However, to ensure high quality results and correct interpretation the respective in vitro assays are currently still reserved for specialized centers with both clinical and laboratory expertise in the management of DHR. Considering the clinical subtypes, applicable test systems can be subdivided into immediate- and late-type in vitro assays. 

## Immediate-type in vitro assays 

### Detection of drug-specific IgE antibodies 

Serum-specific IgE testing is a safe, in vitro diagnostic tool for the identification of immediate-type drug reactions. At present, however, validated tests for detecting specific IgE in serum are available only for a limited number of drugs (mainly β-lactam antibiotics) [2]. The diagnostic value of specific serum IgE measurements is limited not only by the small number of drugs for which such tests are available, but also by their highly variable sensitivity and specificity. A negative specific IgE result does not necessarily rule out an IgE-mediated drug allergy and therefore has only limited negative predictive value. As the retrieval of positive results is time dependent, it is recommended to perform these analyses as soon as possible after the index reaction. For example, it has been shown that in one-third of patients, drug-specific IgE antibodies were no longer detectable 6 months after an anaphylactic reaction to penicillin, while after 12 months, positive concentrations were found in only half of them [3]. Another, more general problem of low sensitivity of specific IgE assays may be still linked to the lack of knowledge about the allergenic determinants of the respective drugs used in these test systems [4]. 

### Detection of drug-reactive effector cells: basophil activation test 

Drug-dependent activation of basophils – as easily accessible immediate-type effector cells – can be measured either by the release of preformed (e.g., histamine) and newly synthesized mediators (e.g., leukotrienes) through histamine release assays and the cellular antigen stimulation test (CAST or CAST-ELISA (enzyme-linked immunosorbent assay)), respectively, or by the upregulation of specific activation markers, i.e., CD63 or CD203c, on their cell surface utilizing the basophil activation test (BAT) [5, 6]. The BAT is the most-established cellular in vitro test system for assessing immediate-type drug reactions. According to the guidelines, for patients with anaphylactic reactions of grade II or higher, in vitro diagnosis using BAT is recommended over in vivo drug challenge tests for safety reasons [2]. However, sensitivities are not that high accounting in general for around 50 – 60% [6, 7]. For β-lactam antibiotics, the rates are even lower, ranging from 20 to 50% [8, 9, 10]. This means that negative results do not rule out drug sensitization and should therefore be confirmed by other tests, like skin or provocation tests whenever there are no concerns regarding patient safety. The specificity, on the other hand, ranges from about 78 to 100%, which implies that positive test results, in combination with the corresponding medical history, have a high predictive value [11]. 

Nevertheless, the BAT has some inherent limitations. One problem, for example, is the relatively high non-responder rate of 6 to 20%, i.e., the proportion of basophils that do not upregulate CD63 and CD203c (or release leukotrienes and cytokines) in response to IgE-dependent stimulation [12]. Furthermore, the results – and consequently the sensitivity and specificity – may vary depending on the respective drug (including the utilization of metabolites as potential elicitors) or its concentration used for activation and the specific test protocol. However, efforts are increasingly being made to standardize not only the laboratory protocols but also flow cytometer settings, the management of drugs, and the consistent interpretation of results (e.g., establishment of the thresholds) [13, 14]. Similar to IgE antibodies, positive results are most reliably detected during the first 1 – 3 years. Therefore, such testing should ideally be performed within 2 weeks and 12 months after the drug-induced immediate reaction [11]. 

Due to the low sensitivity of CAST [15, 16] and BAT, and their inconsistent and insufficient diagnostic performance in non-IgE-mediated reactions, particularly in patients with Cox-I/II-inhibitor (NSAID) hypersensitivity [5], the use of these in vitro tests for the routine diagnosis of DHR is repeatedly called into question [17]. Consequently, there are ongoing efforts to establish new in vitro test approaches that circumvent these limitations. 

### Detection of drug-reactive effector cells: mast cell activation test 

In recent years, mast cell-based assays, particularly the mast cell activation test (MAT) and its variant, the passive MAT (pMAT), have emerged as promising alternatives. In IgE-mediated DHR, the MAT has shown encouraging results as mast cells can be passively sensitized with patient serum containing drug-specific IgE. Despite all its apparent limitations [18], the MAT demonstrates good concordance with BAT and skin testing, with reported sensitivities of up to approximately 70 – 75% and very high specificity, particularly for β-lactam antibiotics and perioperative allergens [19, 20]; consequently, it may be especially valuable in cases with negative BAT or inconclusive in vivo testing. 

In contrast, the role of MAT in non-IgE-mediated (pseudoallergic) drug reactions remains poorly defined. While mast cells can be activated via non-IgE-dependent mechanisms – such as direct activation through receptors like MRGPRX2 – current MAT protocols are primarily optimized for IgE-mediated pathways. Consequently, evidence supporting the use of MAT in NSAID hypersensitivity and other pseudoallergic reactions is rather sparse, and its diagnostic sensitivity and specificity in this context remain unclear [18]. Across all immediate-type in vitro assays, inconsistent and clinically difficult-to-interpret results still remain a significant challenge. Even though the BAT and, especially, the MAT still have optimization potential [21], controlled drug provocation testing (i.e., in patients for whom it is appropriate) remains the diagnostic gold standard, and in vitro assays such as BAT and increasingly MAT should therefore be regarded still as complementary tools. 

### Biomarker for drug-induced anaphylaxis: mast cell tryptase 

Tryptase is a serine protease encompassing several isoforms. Among these, β-tryptase is released during mast cell activation, resulting in vasodilatation and bronchial hyperresponsiveness, thus serving as a marker for anaphylaxis [22, 23]. Commercially available assays measure total tryptase without differentiating between α-protryptase and β-tryptase. Total serum tryptase concentration reaches its peak 1 – 2 hours after the onset of an immediate-type DHR, returning to baseline levels approximately 24 – 48 hours later [24]. High levels are associated with an increased reaction severity [25]. A relevant mast cell activation can be assumed if the serum tryptase concentration, measured about 1 – 2 hours after a DHR, is at least equivalent to 1.2 × (individual) baseline tryptase concentration + 2 ng/mL [2]. A pooled sensitivity of 62% and specificity of 83% has been demonstrated in a recent meta-analysis utilizing this formula [26]. Analyzing anaphylactic perioperative DHR, sensitivity was 78% and specificity 91%, however, the negative predictive value amounted to only 44%, indicating that a substantial proportion of patients do not exhibit a detectable rise in tryptase [27]. Other biomarkers of anaphylaxis under consideration include platelet-activating factor (PAF), the enzyme PAF acetylhydrolase, urinary prostaglandin D2, and combined measurement of tryptase and histamine [23]. The clinical practicability and availability of these markers remain limited, necessitating further studies [26]. Currently, serum tryptase remains the most cost-effective and widely used diagnostic marker in clinical practice, with elevated concentrations pointing to an immediate-type DHR. 

## Late-type in vitro assays 

As T cells play a central role in the pathogenesis of late-type DHR [28], they are the focus of different in vitro test systems aiming to identify the culprit drug in these reactions [29]. 

### Lymphocyte transformation test 

The lymphocyte transformation test (LTT) measures the proliferation index of T cells among peripheral blood mononuclear cells labeled with a radioactive marker when incubated with the drug [30]. The LTT is probably the currently most exactly standardized late-type assay regarding read-out specifications [31]. Ideally, cells should be retrieved 2 – 6 weeks after the DHR. Earlier measurements might be affected by nonspecific bystander activation that is still present, while at later time points false-negative results might occur, although for drug rash with eosinophilia and systemic symptoms (DRESS) positive results delayed by several months have been reported as well [32, 33]. Results can be drug-dependent as ibuprofen and sulfamethoxazole have been shown to elicit unstable false-positive T-cell proliferation [34]. 

### Enzyme-linked immunospot assay 

The enzyme-linked immunospot (ELISPOT) assay determines drug-induced T-cell functional changes measuring cytokine secretion of individual T cells upon incubation with the implicated drug [29, 35]. Different cytokines (IFN-γ, TNF-α, IL-2, -4, -5, -8, -13, -17, -22, granzyme B) can serve as targets, with IFN-γ representing the best-established and probably most reliable marker, but some others may be better suited for examining specific DHR, like IL-4, -5, -13 for DRESS or sometimes maculopapular exanthema and granzyme B for SJS/TEN [31, 36, 37, 38]. Combination of different cytokines could enhance the detection rate [36, 39]. As the assay depicts single cytokine-secreting cells, it allows to detect even very low numbers of drug-specific T cells which account for only 0.01 – 0.08% of all circulating T cells [40, 41]. Increased likelihood of identifying the eliciting drug in samples taken during the acute DHR phase has been reported for the ELISPOT assay, potentially due to loss of drug-specific memory T cells or their migration from the circulation to the skin over the course of recovery [42, 43]. Thus, (an initial) sampling during the first 30 days after onset of the DHR seems reasonable [36]. In bullous DHR, blister fluid could contain increased numbers of pathogenic T cells and thus serve as an additional source for analysis in the acute phase [44]. 

### Activation-induced marker assay 

Upon incubation of peripheral blood cells with a DHR-eliciting drug, activation markers such as CD25, CD69, CD71, CD137 or CD154 are upregulated on the surface of drug-specific T cells and can thus be measured by flow cytometric analysis [42, 45, 46, 47, 48, 49]. As the cells become activated relatively quickly, in comparison to the other assays less time of about 16 – 72 hours instead of 7 – 10 days is required for retrieving results. However, expression of the marker can be inconsistent and may vary depending on the responsible drug or the underlying DHR reaction, making the test less reliable and necessitating the evaluation of further parameters, which qualifies activation-induced marker (AIM) rather as a supporting additional than a primary diagnostic in vitro test [29, 42]. 

### Cytokine detection assays: enzyme-linked immunosorbent assay, flow cytometry-based bead assay, multiplex bead-based immunoassay 

In contrast to the former assays, this assay does not determin drug-specific cells; instead, it quantifies the amount of one or several cytokines, like IL-2, -4, -5, -10, -13, IFN-γ, granulysin (in SJS/TEN patients), granzyme B, secreted into the supernatants of cultured peripheral blood cells. Studies utilizing these in vitro tests in the investigation of DHR have shown varied results, without significantly substantiating their utility as a diagnostic tool so far [31, 50]. 

### General aspects and comparative assessment 

Among the applicable test systems for analyzing late-type DHR, LTT and ELISPOT are currently the most comprehensively investigated and utilized assays for in vitro diagnosis of late-type DHR. While sensitivity and specificity of these tests can vary widely among different studies, thorough analyses have shown sensitivities around 50 – 60% for both assays [34, 36, 37, 50, 51, 52, 53, 54]. Percentages are higher than those found for patch tests in comparative surveys, although on the other hand not all patients with a positive skin test react in the in vitro assays [34, 54, 55]. They may differ depending on the examined drug (for example, they do not seem to be useful in the diagnosis of late-type DHR to iodinated contrast media [56, 57, 58]) or the investigated DHR with in particular slightly lower rates for TEN. The specificity is mostly high with rates of above 80 – 90%, positive predictive values lie around 80 – 100% and negative predictive values around 50 – 80% [50]. Modifications to increase the detection rates include the suppression of inhibitory influences on T-cell activation (for example, addition of anti-PD-L1 antibody or depletion of regulatory T cells) or enhancement of T-cell activation through stimulatory molecules (for example, IL-2, anti-CD3/CD28, phytohemagglutinin, IL-7/IL-15), although the effects are often not strong and may be achieved at the expense of specificity [53, 59, 60]. Another measure could be the inclusion of drug metabolites, which in some case might be the relevant antigen [61, 62]. 

Regarding the patient, the timing of sample collection is critical and should be attempted rather soon after recovery from the DHR. Recommendations vary between 2 – 12 weeks, with analysis of blood cells from patients with acute generalized exanthematous pustulosis (AGEP) and SJS/TEN after about 4 and DRESS after 12 weeks [31, 36]. However, positive results may be also obtained many years to decades later [34, 39, 41]. The ELISPOT assay can (and probably should) be performed very early with increased sensitivity, even during the acute phase of the DHR [36, 43, 52]. Of note, if the patient is (still) under immunosuppressive treatment with corticosteroids, this should not interfere with the tests’ reliability if the dose does not exceed 0.2 mg prednisolone equivalent per kg body weight [29]. 

## Conclusion: current status, challenges, and future perspectives 

In summary, cellular in vitro assays could serve as helpful tools in diagnosis of late-type DHR. Currently, the LTT and ELISPOT assay would be considered as the most appropriate tests with reasonable sensitivity and high specificity. Utilization of two assays or evaluation of more than one parameter in one test system (for example, several cytokines like IFN-γ, IL-5 and granzyme B by ELISPOT) will increase the detection rate [36, 43, 63]. Thus, especially for severe late-type DHR like AGEP, DRESS and SJS/TEN, where drug provocation tests are not an option, one of these assays should be considered to enhance the likelihood of identifying the culprit drug that must subsequently be avoided [52, 55]. However, for proper regular use, standardized protocols

- addressing the handling and analytical processing of samples, utilized drug formulations and concentrations,
- determining the required, appropriate controls,
- specifying uniform assessment criteria, including cut-off thresholds as well as features defining a positive/negative result,
- and considering intra- and interindividual variability

have to be established and agreed upon. In addition, more data on pediatric populations are needed to include this vulnerable group of patients. 

## Pharmacogenetic approaches: human leukocyte antigen typing 

Beyond in vitro assays aiming to identify culprit drugs, pharmacogenetic approaches may help to predict and prevent severe DHR prior to drug exposure. Specific human leukocyte antigen (HLA) alleles have been associated with an increased susceptibility to severe T-cell-mediated adverse drug reactions, such as SJS, TEN, and DRESS [64]. Distinct HLA molecules are thought to facilitate aberrant presentation of drugs or drug-derived antigens to T cells, thereby triggering pathological immune activation [65]. Thus, certain HLA variants may present a strong genetic risk factors for a DHR, although they are not sufficient for disease development [64]. 

A prominent example is the strong association between HLA-B*57:01 and abacavir-induced DHR, while HLA-B*15:02 and HLA-B*58:01 have been linked to carbamazepine- and allopurinol-induced severe cutaneous adverse reactions, respectively [66, 67, 68]. Current evidence supports a targeted rather than universal pre-emptive HLA screening strategy prior to initiation of selected high-risk drugs. Routine HLA-B*57:01 testing before abacavir treatment is strongly recommended due to its high predictive value and established clinical benefit, either by PCR-based assays or next-generation sequencing from peripheral blood samples [69]. HLA-B*15:02 screening should be considered before carbamazepine initiation, particularly in patients of Asian ancestry, whereas HLA-B*58:01 testing may be beneficial in high-risk patients receiving allopurinol, especially those with chronic renal insufficiency or populations with increased allele prevalence. However, broad HLA screening before any drug administration is currently not recommended due to the overall low positive predictive value and rarity of many severe DHR [70]. 

## Concluding remarks 

Immediate- and late-type reaction in vitro tests can provide additive information in the diagnostic workup of selected cases of DHR. Measurement of serum mast cell tryptase in patients suffering from anaphylaxis after drug intake can be of value determining if a drug-specific IgE-mediated reaction occurred. For identification of the eliciting drug, so far, besides IgE analysis of anaphylaxis against β-lactam antibiotics, in vitro assays are still second choice to skin tests, which are easy to perform with drugs at hand, directly yielding results [2], as well as to drug provocation tests (if indicated and safe). However, drug challenge may not be feasible due to individual circumstances of the patient (for example, due to pregnancy, severity of the potentially elicited DHR (like SJS, TEN, or DRESS) or co-morbidities), missing (infrastructural) options for performing and medically supervising the challenge or refusal by the patient. In these cases, certain in vitro assays may be of help in identifying a culprit drug to be avoided in the future (Figure 1). Notably, these assays cannot replace clinical history. However, some in vitro assays have shown reasonably high sensitivity and specificity compared to skin tests as has been demonstrated in comparative studies for the LTT and ELISPOT assay [34, 54, 55]. Nevertheless, tests like the CAST, histamine release, AIM, and cytokine detection systems currently cannot be regarded as helpful tools in the diagnosis of DHR, largely due to the lack of validation and standardization. Further refinements considering the handling and processing of test samples (for example, the MAT as an immediate-type reaction test requiring only patient serum) as well as amended standardization are still needed to strengthen arguments for their future utilization. 

## Authors’ contributions 

CM, SCHL, VH, and WP contributed to preparation, writing, and proofreading of the manuscript. 

## Funding 

No funding received. 

## Conflict of interest 

CM has received a travel grant from Phadia AB/Thermo Fisher Scientific, and personal fees and a research grant from Almirall. 

SCHL has received personal fees from Almirall and has been a speaker for Sanofi. 

WP has received personal fees and/or research grants from ALK-Abelló, Almirall, Janssen-Cilag, Leo Pharma, Novartis, Sanofi, and Phadia AB/Thermo Fisher Scientific. 

VH has no conflict of interest. 

**Figure 1.**
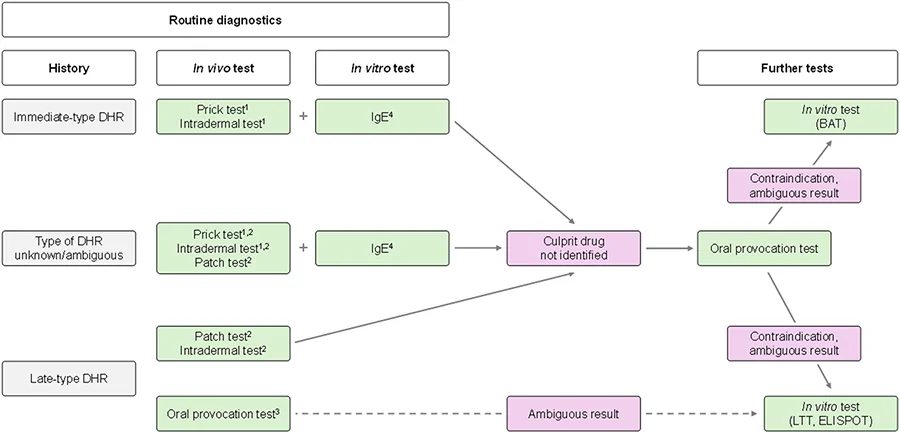
Diagnostic workup: identification of culprit or alternative, tolerated drug(s) in patients with drug hypersensitivity reaction (based on expert recommendations of [2]). Tests are chosen and performed according to clinical history, ideally 4 weeks to 6 months after the reaction. If routine diagnostics allow no clear diagnosis, oral provocation testing is recommended. In case of contraindications, lacking possibility, ambiguous result, or refusal of the patient, appropriate in vitro assays (as indicated), performed in a specialized center should be considered. ^1^Immediate reading after 20 minutes; ^2^delayed reading after 2 and 3 days; ^3^in children with “mild rash”, direct oral challenge can be considered; ^4^available only for a few β-lactam antibiotics.
